# Flexible Bronchoscopy in Neonates With Congenital Diaphragmatic Hernia

**DOI:** 10.1002/ppul.71128

**Published:** 2025-05-14

**Authors:** Judith Leyens, Lukas Schroeder, Carmen Salatsch, Joachim Schmitt, Hemmen Sabir, Andreas Mueller, Florian Kipfmueller, Till Dresbach

**Affiliations:** ^1^ Department of Neonatology and Pediatric Intensive Care University Hospital of Bonn Bonn Germany; ^2^ Division of Neonatology, Department of Pediatrics, BC Women's and Children's Hospital University of British Columbia Vancouver Canada; ^3^ Department of Neonatology University Children's Hospital Mannheim, University of Heidelberg Mannheim Germany

**Keywords:** bronchoscopy, congenital diaphragmatic hernia, extracorporeal membrane oxygenation, flexible bronchoscopy, plastic bronchitis

## Abstract

**Background:**

Flexible bronchoscopy (FB) may facilitate ECMO and ventilator weaning through diagnosing airway anomalies and removal of mucous plugs in the critically‐ill pediatric population. Only few studies on FB in critically‐ill neonates exist, and even fewer focus on neonates with congenital diaphragmatic hernia (CDH) requiring extracorporal membrane oxygenation (ECMO). This study aims to evaluate the risk and benefit of FB in infants with CDH.

**Methods:**

A retrospective review of CDH infants treated at a specialized single center between October 2019 and August 2024 was conducted. Baseline characteristics were compared between patients with and without FB. Procedural indications, findings and complications were analyzed.

**Results:**

A total of 142 newborns were analyzed. Infants requiring FB (*n *= 29, 20.4%) exhibited an overall higher disease burden (lower observed‐to‐expected lung‐to‐head ratio [*p* < 0.001), liver herniation (*p* = 0.002), ECMO (*p* < 0.001), defect size (*p* = 0.042), congenital anomalies (*p* = 0.019), heart defects (*p* = 0.010)]. The primary indications for the total 56 FBs were prolonged weaning and pulmonary hemorrhage. The most common complication was self‐resolved hypoxemia (16.1%). Bronchial casts were found in 31.6%. Tracheo‐/bronchomalacia was diagnosed in 16 infants (55.2%). Postprocedural chest Xrays were mostly unchanged (61.9%). A trend to achieve higher tidal volumes post‐FB (*p* = 0.090) with similar peak inspiratory pressures (*p* = 0.917) was noted.

**Conclusions:**

In critically‐ill neonates with CDH, FB was safe, with a high diagnostic and potential therapeutic yield. The necessity for FB may be an additional indicator of CDH disease severity. Further research is needed to establish uniform assessment metrics and explore other modalities such as electrical impedance tomography or lung ultrasound in the context of FB.

**Abbreviations**
ACT: Activated clotting time
BAL: Bronchoalveolar lavage
CDH: Congenital diaphragmatic hernia
CDHSG: CDH study group
CHD: Congenital heart defects
ECMO: Extracorporeal membrane oxygenation
EIT: Electrical impedance tomography
FB: Flexible bronchoscopy
FETO: Fetoscopic endoluminal tracheal occlusion
IQR: Interquartile range
LUS: Lung ultrasound
NICU: Neonatal intensive care unit
o/e LHR: Observed‐to‐expected lung‐to‐head‐ratio
PICU: Pediatric intensive care unit
PTT: Partial thromboplastin time
TFLV: Total fetal lung volume

## 1 Introduction

Congenital diaphragmatic hernia (CDH) affects approximately 2 in 10.000 infants [1]. Disease severity is associated with antenatal markers (liver position, observed‐to‐expected lung‐to‐head‐ratio (o/e LHR), total fetal lung volume (TFLV)), pulmonary hypoplasia and cardiac dysfunction, as well as associated comorbidities, especially congenital heart defects (CHD) [2]. Fetoscopic endoluminal tracheal occlusion (FETO) has been shown to improve survival for severe left‐sided CDH [3]. The risks of FETO include premature rupture of membranes and delivery, as well as airway anomalies such as tracheo‐ and bronchomalacia [4]. Approximately one‐third of CDH neonates are offered extracorporeal membrane oxygenation (ECMO) after birth, and thus should be preferably delivered in proximity to specialized neonatal/pediatric intensive care units (NICU/PICU) [5]. Complications of ECMO may be secondary to cannulation or systemic anticoagulation, including thrombosis and bleeding, with neonates being especially prone to intracerebral, gastrointestinal, and pulmonary hemorrhage. Infants with CDH are thus at high risk of severe pulmonary disease. Flexible bronchoscopy (FB) is a validated, useful bedside tool to diagnose upper and lower airway abnormalities [6]. It also provides therapeutic advantages through airway clearance and removal of bronchial casts via bronchoalveolar lavage (BAL), as well as local bleeding control and drug instillation [7]. Anatomical airway anomalies and pulmonary hemorrhage are not uncommon in neonates with CDH, which may be secondary to the primary disease or treatment complications [8]. Therefore, neonates with CDH may present a subgroup of NICU/PICU patients that require and benefit from FB the most [8]. Possible complications of FB are postinterventional fever, hemorrhage, and pneumothorax, but FB has been shown to be an overall safe procedure even among ECMO patients [9, 10]. Most studies on FB in the pediatric population focus on patients beyond the neonatal age, with only few studies in neonates, and even less in neonates with ECMO and/or CDH [11, 12]. The aim of this study is to report our single center experience with neonatologist‐provided FB in CDH neonates including data on patient selection, indication for FB, safety, diagnostic, and therapeutic yield.

## 2 Methods

### 2.1 Study Design

**Figure 1:**
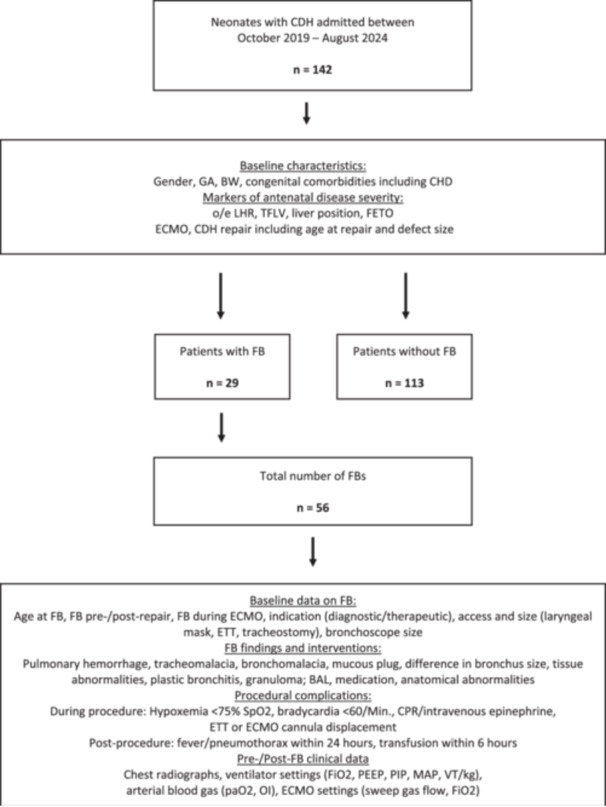
Overview of retrospective study design. Baseline characteristics were obtained for all admitted CDH infants. The total number of all bronchoscopies in all infants with CDH and FB was collected and further analyzed. BAL, bronchoalveolar lavage; BW, birth weight; CDH, congenital diaphragmatic hernia; CHD, congenital heart disease; CPR, cardiopulmonary resuscitation; ECMO, extracorporeal membrane oxygenation; ETT, endotracheal tube; FiO2, fraction of inspired oxygen; FB, flexible bronchoscopy; FETO, fetoscopic endoluminal tracheal occlusion; GA, gestational age; MAP, mean airway pressure; o/e LHR, observed to expected lung‐to‐head‐ratio; OI, oxygenation index; paO2, arterial partial pressure of oxygen; PEEP, positive end‐exspiratory pressure; PIP, peak inspiratory pressure; TFLV, total fetal lung volume; VT, tidal volume.

An overview of our study design is shown in Figure 1. We performed a retrospective cohort study of all infants with CDH who received postnatal care at the Department of Neonatology and Pediatric Intensive Care at the University Hospital of Bonn, Germany between October 2019 and August 2024. We obtained data providing baseline information on patients' demographics. Defect sizes were staged according to the CDH study group (CDHSG) classification. Associated CHDs were classified in three groups: (A) nonsignificant (e.g. isolated atrial septal defect, small ventricular septal defect, peripheral pulmonary stenosis, nonsignificant ductus arteriosus), (B) significant ductus arteriosus defined as requiring interventional/surgical closure, or (C) significant CHD requiring early intervention/surgery (e.g. duct‐dependent). The total number of FBs was analyzed regarding baseline information on timing, indication, procedural circumstances, findings, interventions, and complications as demonstrated in Figure 1. Indication for FB was defined as diagnostic if an anatomical airway anomaly was suspected without intent for BAL, and therapeutic if BAL was intended for atelectasis, hemorrhage, or query infection. To standardize data analysis, pre‐ and post‐FB data on ventilator and ECMO settings were obtained at 6 AM on the day of and 6 AM the day after FB. Periprocedural chest radiographs 24 h before and after FB, if available, were reviewed by two physicians (JL/TD), and rated as either improved (improvement in lung aeration and/or reduction of atelectasis), unchanged, or worsened. Delivery room FBs that were performed in FETO patients with the sole indication to retrieve remaining intratracheal balloons were excluded. For the purpose of this study, bronchoscopy reports and images were systematically reanalyzed by two physicians with substantial experience in FB (JL/TD). Data were collected from the electronic medical records (ICM‐PDMS, Draeger Medical Germany GmbH, Lübeck, Germany; Neodat, Paedsoft, Tübingen, Germany; Orbis, Dedalus Healthcare GmbH, Bonn, Germany). This study followed the Declaration of Helsinki guidelines and has been approved by the University Hospital of Bonn Institutional Ethics Committee (No. 2024‐435‐BO). Informed consent was waived due to the retrospective study design.

### 2.2 CDH and ECMO Treatment Protocol

Perinatal management of CDH, delivery room resuscitation, and postnatal management including indication for ECMO were conducted according to the CDH Euro Consortium Consensus updated 2015 guidelines [13]. Our institution offers FETO according to the previously published guidelines [3]. Our institutional standards are nasotracheal intubation and venovenous ECMO. Cannulation is routinely performed by trained neonatologists using ultrasound‐guided Seldinger technique as previously published [14]. For anticoagulation during ECMO, unfractionated heparin and antithrombin are used with the following goals: ACT 180–200 s, PTT 60–80 s, Anti‐Xa 0.4–0.7, Antithrombin 80–120%, Fibrinogen > 150 mg/dl. Transfusion goals are hemoglobin > 10 g/dl and platelets > 80 G/l.

### 2.3 Flexible Bronchoscopy Protocol

All FBs were performed at bedside. Most procedures were performed by one or two trained neonatologists (JL, AM, TD) with considerable experience within the setting of high‐risk procedural circumstances. A second neonatologist or experienced neonatal/critical care fellow provided adequate periprocedural anesthesia as per institutional standards. Pediatric flexible bronchoscopes from either Olympus (BF‐N20 Fibre Bronchoscopes, Olympus Corporation, Tokyo, Japan), Storz (Broncho‐Fiberscope Karl Storz SE & Co. KG, Tuttlingen, Germany), or Ambu (Ambu® aScope™ 5 Broncho, Ambu A/S, Ballerup, Denmark) were used.

### 2.4 Statistical Analysis

Categorical variables are reported as absolute number n with percentages (%). Continuous variables are reported as median and interquartile range (IQR). Numerical variables are reported as mean and standard deviation. Infants with CDH with and without FB were compared using Fisher's exact test, and independent samples t‐test or paired samples t‐test, as appropriate. A *p*‐value of < 0.05 was considered statistically significant. For statistical analyses, IBM SPSS Statistics (IBM Corp. Released 2023. IBM SPSS Statistics, Version 29.0.2.0 Armonk, NY: IBM Corp) was used.

## 3 Results

A comprehensive overview of the CDH infants baseline data, and comparison of infants with and without FB is demonstrated in Table 1. Antenatal data on o/e LHR and TFLV were available for 122 (85.9%) and 90 (63.4%) patients, respectively. Disease burden was significantly more severe in infants with FB, as evidenced by a lower o/e LHR (*p* < 0.001), higher rate of intrathoracic liver herniation (*p* = 0.002), FETO (*p* = 0.015), ECMO (*p* < 0.001), CDH repair during ECMO (*p* < 0.001), later CDH repair (0.001), larger defect size (*p* = 0.042), more infants with non‐isolated CDH (*p* = 0.019), and CHD (*p* = 0.010). Accordingly, overall mortality was significantly higher in infants with FB (*p* < 0.001).

**Table 1:** Overview of patients' characteristics for overall cohort, and for patients with and without flexible bronchoscopy (FB). Results are reported as absolute numbers (n) with %, or mean with standard deviation. A *p*‐value < 0.05 is considered statistically significant and marked *.

	All infants	FB	No FB	p‐value
	*n* = 142	*n* = 29 (20.4%)	*n* = 113 (79.6%)	
Antenatal Diagnosis (*n*=)	124 (87.3%)	25 (86.2%)	99 (87.6%)	0.839
Left‐sided CDH (*n*=)	123 (86.6%)	26 (89.7%)	97 (85.8%)	0.798
Liver‐up (*n*=)	81 (57%)	24 (82.8%)	57 (50.4%)	0.002*
o/e LHR (%)	41.2 ± 14.1	32.9 ± 11.0	43.4 ± 14.1	< 0.001*
TFLV (%)	38.5 ± 18.2	32.4 ± 15.8	40.5 ± 18.6	0.065
FETO (*n*=)	23 (16.2%)	9 (31.0%)	14 (12.4%)	0.015*
Female (*n*=)	72 (50.7%)	13 (44.8%)	59 (52.2%)	0.478
GA (weeks+days)	36 + 6 ± 17	36 + 3 ± 16	37 + 0 ± 18	0.286
BW (kg)	2.8 ± 0.7	2.7 ± 0.7	2.9 ± 0.7	0.274
ECMO	52 (36.6%)	20 (69.0%)	32 (28.3%)	< 0.001*
CDH repair	130 (91.5%)	27 (93.1%)	103 (91.2%)	0.736
Repair on DOL	5.8 ± 2.6	7.6 ± 2.4	5.4 ± 2.5	< 0.001*
Repair during ECMO	12 (9.2%)	8 (27.5%)	4 (3.6%)	< 0.001*
Defect Size				0.042*
A	13 (10.0%)	‐‐‐	13 (12.6%)	
B	39 (30.0%)	4 (14.8%)	35 (33.9%)	
C	49 (37.7%)	14 (51.9%)	35 (33.9%)	
D	29 (22.3%)	9 (33.3%)	20 (19.4%)	
Non‐isolated CDH	35 (24.6%)	12 (41.4%)	23 (20.4%)	0.019*
CHD	31 (21.8%)	12 (41.4%)	19 (16.8%)	0.010*
… (A)	12 (38.7%)	3 (25.0%)	9 (47.3%)	
… (B)	16 (51.6%)	7 (58.3%)	9 (47.3%)	
… (C)	3 (9.7%)	2 (16.6%)	1 (5.4%)	
Early mortality (< 24 h)	9 (6.3%)	1 (3.4%)	8 (7.1%)	0.474
Overall mortality	39 (27.5%)	18 (62.1%)	21 (18.6%)	< 0.001*

Abbreviations: BW, birth weight; CDH, congenital diaphragmatic hernia; CHD, congenital heart disease; DOL, day of life; ECMO, extracorporeal membrane oxygenation; FB, flexible bronchoscopy; FETO, fetoscopic endoluminal tracheal occlusion; GA, gestational age; o/e LHR, observed to expected lung‐to‐head‐ratio; TFLV, total fetal lung volume.

**Figure 2:**
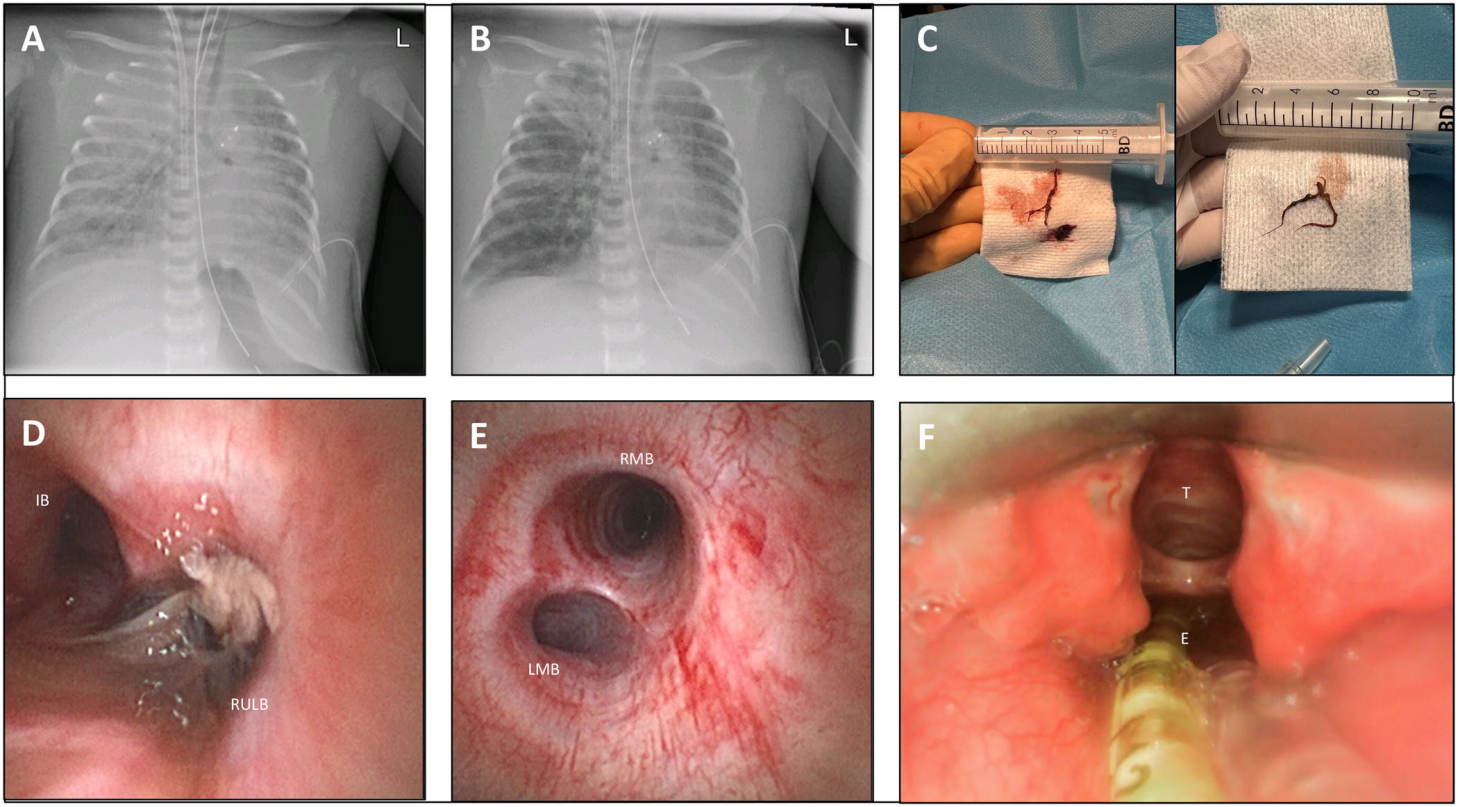
Pre‐(A) and post‐(B) flexible bronchoscopy chest radiographs, demonstrating resolution of the complete opacification of the right upper lobe and improved aeration of both lungs after the removal of bronchial casts (C). (D) Bronchial cast completely obstructing the RULB. (E) In this patient with left CDH, the LMB appeared smaller than the right main bronchus RMB. (F) Accidental finding of a laryngeal cleft type II during flexible bronchoscopy. E: esophagus; IB: intermediate bronchus; LMB: left main bronchus; RMB: right main bronchus; RULB: right upper lobe bronchus; T: trachea. [Color figure can be viewed at wileyonlinelibrary.com]

A total of 56 FBs were performed in 29 infants with CDH. A median of one FB was performed per patient (IQR 1–3). Before CDH repair, 10 FBs (17.9%) were performed in six patients (20.6%), and post‐CDH repair 46 FBs (82.1%) in 23 (79.3%) patients. Pre‐repair FBs were performed at a median of 7.8 days (IQR 5,6–10.3) days before CDH repair at a median age of 4.7 days (IQR 1.8–10.3). The most common indication for pre‐repair FBs was pulmonary hemorrhage in the context of ECMO (*n* = 8, 80%). Post‐repair FBs were performed at a median of 27.3 days (IQR 9.6–42.2) after CDH repair at a median age of 33.5 days (IQR 15.9–47.4). The most common indication for post‐repair FB was BAL for prolonged weaning from invasive ventilation and/or ECMO (*n* = 22, 47.8%), followed by pulmonary hemorrhage in the context of ECMO (*n* = 13, 28.2%). Table 2 offers additional periprocedural information. One FB (1.7%) was performed with a 1.8 mm outer diameter BF‐N20 Fibre Olympus Bronchoscope, 35 (62.5%) with a Storz bronchoscope (Broncho‐Fiberscope 2.8 × 54, outer diameter 2.9 mm), and since November 2023, all FBs (*n* = 20, 35.7%) were performed with a 2.7 mm outer diameter Ambu bronchoscope (Ambu® aScope™ 5 Broncho 2.7/1.2). Endotracheal tube or tracheostomy sizes were available for 51 (91.1%) FBs, with 3.5 mm being the most common size (*n* = 47, 83.9%), followed by 4.0 mm (*n* = 3, 5.4%), and 4.5 mm (*n* = 1, 1.8%). Out of the 47 FBs with therapeutic indication, the most common indication was BAL for prolonged weaning (*n* = 24, 51.1%), followed by pulmonary hemorrhage in the context of ECMO (*n* = 19, 40.4%), and pulmonary hemorrhage without ECMO (*n* = 4, 8.5%). Pre‐ and postprocedural chest radiographs were available for 42 (75.0%) procedures, and evaluated as unchanged in 26 (61.9%), worsened in 2 (4.8%), and improved in 14 cases (33.3%) (Figure 2A, B). Bronchial casts were present in 31.6% (Figure 2C, D). A difference between main bronchial sizes was seen in 78.9% (Figure 2E). Tracheo‐/bronchomalacia was diagnosed in a total of 33 FBs (58.9%), or 16/29 infants (55.2%). Significant additional anatomical findings were found in two patients: one with a laryngeal cleft type II (Figure 2F), and one with left‐sided CDH who passed away in the delivery room who was found to have additional right‐sided main stem bronchus atresia. There was no significant difference in airway anomalies between left‐sided (*n* = 14, 53.8%) and right‐sided CDH (*n* = 2, 66.7%; *p* = 0.58), or bronchial size hypoplasia of the affected side (*n* = 19, 86.4% vs. *n* = 2, 66.7%; *p* = 0.422), respectively.

**Table 2:** Overview of indications, findings, periprocedural circumstances, and complications of all performed FBs.

Patients with FB (*n* = 29)	Total FBs (*n* = 56)
FB during ECMO (*n*=)	30 (53.6%)
Indication (*n*=)	
Diagnostic	9 (15.8%)
Therapeutic	47 (82.5%)
Findings/Intervention	
Pulmonary hemorrhage	
None (*n*=)	30 (52.6%)
Without ECMO (*n*=)	5 (8.8%)
With ECMO (*n*=)	21 (36.8%)
Tracheomalacia (*n*=)	15 (26.3%)
Bronchomalacia (*n*=)	18 (31.6%)
Mucous plug atelectasis (*n*=)	38 (66.7%)
Primarily affected CDH side	
Findings on CDH side (*n*=)	12 (21.1%)
Findings on non‐CDH side (*n*=)	3 (5.3%)
Bilateral findings (*n*=)	38 (66.7%)
Difference in main bronchial sizes (*n*=)	45 (78.9%)
Abnormal mucous membrane (*n*=)	52 (91.2%)
Bronchial casts/plastic bronchitis (*n*=)	18 (31.6%)
Granuloma (*n*=)	1 (1.8%)
BAL (*n*=)	43 (75.4%)
Intrabronchial drug application (*n*=)	4 (7%)
Access (*n*=)	
Laryngeal mask	3 (5.3%)
ETT	48 (84.2%)
Tracheostomy	4 (7%)
Complications	
Transfusion (*n*=)	15 (26.8%)
Hypoxemia (*n*=)	9 (16.1%)
Bradycardia (*n*=)	1 (1.7%)
CPR/IV epinephrine (*n*=)	0 (0%)
ETT displacement (*n*=)	1 (1.7%)
ECMO cannula displacement (n=)	0 (0%)
Pneumothorax (*n*=)	0 (0%)
Postinterventional fever (*n*=)	0 (0%)

Abbreviations: BAL, bronchoalveolar lavage; CPR, cardiopulmonary resuscitation; ECMO, extracorporeal membrane oxygenation; ETT, endotracheal tube; FB, flexible bronchoscopy.

In 14/15 cases requiring transfusion within 6 h of FB, FB was performed during ECMO therapy, but upon further review, all of these cases already required frequent transfusion before FB, and no bleeding complications from FB were noted. Periprocedural pulmonary hemorrhage was only reported in one of these cases, being a neonate with severe left‐sided CDH with FETO, a prolonged ECMO course, and plastic bronchitis. One patient without ECMO was electively transfused within 6 h of FB unrelated to the procedure. All episodes of hypoxemia and bradycardia were brief and self‐resolved by simply retracting the bronchoscope.

Table 3 demonstrates data on the ventilatory and ECMO settings pre‐ and post‐FB. No significant differences were noticed, although there was a trend to achieve higher tidal volumes post‐FB (*p* = 0.090) without a relevant change in peak inspiratory pressures (*p* = 0.917).

**Table 3:** Overview of ventilatory and ECMO‐management data pre‐ and post‐flexible bronchoscopy. The number of available data are reported as absolute numbers (*n*), other values are reported as mean with standard deviation. A *p*‐value is demonstrated for paired samples t‐test, and *p* < 0.05 would be considered significant.

	Before FB	After FB	p‐value
FiO_2__Vent. (*n* = 54)	0.72 ± 0.24	0.69 ± 0.23	0.226
MAP (*n* = 51)	14.1 ± 4.7	14.1 ± 4.6	0.980
PEEP (*n* = 52)	7.9 ± 4.1	7.7 ± 4.1	0.268
PIP (*n* = 50)	24.7 ± 4.5	24.7 ± 4.6	0.917
VT (ml/kg) (*n* = 47)	3.94 ± 2.48	4.27 ± 2.63	0.090
FiO_2__ECMO (*n* = 29)	0.87 ± 0.21	0.80 ± 0.28	0.214
Sweep gas flow l/min. (*n* = 29)	0.33 ± 0.20	0.28 ± 0.16	0.113
paO_2_ mmHg (*n* = 42)	85.13 ± 27.55	90.80 ± 34.08	0.313
OI (*n* = 42)	15.05 ± 9.5	14.34 ± 12.10	0.711

Abbreviations: ECMO, extracorporeal membrane oxygenation; FB, flexible bronchoscopy; FiO2, fraction of inspired oxygen; MAP, mean airway pressure; OI, Oxygenation index; paO2, partial pressure of oxygen; PEEP, positive end‐expiratory pressure; PIP, peak inspiratory pressure; VT, Tidal volume

## 4 Discussion

Our study demonstrates that neonatologist‐provided FB can be safely and sufficiently utilized in newborns with CDH. We demonstrated a very low rate of procedural complications despite the high‐risk conditions in our critically ill neonatal cohort. In addition, we observed a high diagnostic yield of airway anomalies through FB in infants with CDH, along with a tendency towards therapeutic benefit, as evidenced by increased tidal volumes post‐FB.

In recent years, pediatric FB has been increasingly used, and led to a decline of rigid bronchoscopy accordingly [15]. The technological development of bronchoscopes with an outer diameter as small as 1.8 mm (no working channel) or 2.7 mm (with working channel) have made FB in neonates possible [16]. FB is a useful diagnostic bedside tool to detect structural airway anomalies. In addition, FB may have therapeutic benefits by enhancing ventilation and reducing atelectasis through airway clearance, removal of mucous plugs and bronchial casts, and local bleeding control. Even for foreign body removal, FB has been successfully used, with a potentially lower complication rate than rigid bronchoscopy [17]. Historically, the majority of FB was conducted by pediatric pulmonologists or otolaryngologists, however, an increasing number of neonatal/pediatric intensivists have acquired the necessary expertise to perform FB [18]. Potential benefits of this management are preventing delays from additional consultations, and familiarity of FB providers with neonatal airways and critical periprocedural settings. Most studies on pediatric FB focus on children beyond the neonatal age [11, 12]. In neonates, premature infants with chronic lung disease and prolonged respiratory support, and infants with congenital anomalies account for the majority of patients assessed with FB [19]. FB in the NICU has been recommended in patients in whom resolution of atelectasis may facilitate earlier extubation and lung protective treatment, and for evaluation of anatomical airway anomalies and subglottic stenosis [16]. There is a lack of uniform definitions to assess the diagnostic and therapeutic yield and potential complications of FB. Major complications, usually defined as pneumothorax, major bleeding, or death, have been reported in less than < 2% of pediatric FBs [20]. Self‐resolved hypoxemia is one of the most commonly reported minor complications [21]. Infants under 2 years of age, and patients with syndromic and cardiac disorders have the highest risk of complications [22]. Most studies report neonatal FB to have few complications, and a high diagnostic yield of 74–95% [23].

Children who require FB on ECMO are considered an especially vulnerable and sick patient group with an increased risk of hemorrhage, who however, may benefit the most from the removal of mucous plugs and bronchial casts [7, 18]. In trained hands, FB during ECMO has been shown to be a fast and safe procedure, with a high diagnostic and therapeutic yield [24]. A survey from 2019 among Level IV NICUs offering ECMO in North America reported that 50% would consider using bronchoscopy to facilitate decannulation [25]. Kamat et al. reported that 22% of all PICU patients on ECMO in their large single‐center cohort underwent FB with only minor complications (no pneumothorax, blood tinged secretions in 34.6%) [26]. Similarly, in their cohort of 14 neonatal and pediatric ECMO patients with 19 FBs, Karlson et al. reported no major complications [27]. Another study on FB in pediatric, primarily cardiac ECMO patients reported no major complications [9]. Babhalgaonkar et al.'s cohort of 155 children on ECMO, of which 23% received FB, included three patients with CDH and two FBs in the neonatal period. An overall diagnostic yield of 53% was reported, and in 62% FB was successful in airway clearance [28]. They reported a slightly higher rate of major complications (8%, including pneumothorax), major bleeding in two episodes, and minor self‐resolving bleeding in 25%. Chest radiographs were improved in 33%. Chest radiographs as a potential outcome metric of FB have been reported to be inconsistent, with some studies reporting improvement in only 15% within 72 h [26, 27]. A secondary analysis from a multicenter cohort in the USA found FB to be safe and beneficial for pediatric VV‐ECMO patients. Interestingly, they found a statistically significant increase between pre‐ and postprocedural tidal volumes [10]. While this finding was not significant in our cohort, we demonstrated a trend of higher post‐procedural tidal volumes without a change in peak inspiratory pressures as well.

Only few studies exist on FB in infants with CDH. In a Japanese study from 2000, FB was frequently performed (101 times in 39 patients, mean 2.6 times per patient) [8]. Airway anomalies were more frequent in right sided CDH, whereas in our cohort CDH side was not a significant factor for airway anomalies. One significant difference between both cohorts is the unusually high number of right‐sided CDH patients in the Japanese FB cohort (*n* = 12, 30.8%), whereas our cohort included only three patients (10.3%) with right‐sided CDH. In comparison, our study also demonstrated a higher rate of anatomical airway anomalies (17.9% vs. 55.2%) and bronchial hypoplasia of the affected CDH side (38.4% vs. 78.9%). While Nose et al. published their study in an era before FETO, these patients may be of particular interest for FB, as a systematic review has found a higher incidence of airway anomalies in FETO patients [4]. A retrospective study analyzed 17 patients with CDH on ECMO [29]. Similar to our findings, most FBs (88%) were performed for persistent atelectasis or mucous plugs after CDH repair, but improvement in chest radiographs was more frequent with 87.5%. In contrast to our study, all patients were extubated and ECMO support increased during the procedure; this difference might be explained by the publication time of this study (2002), and since significant improvement in neonatal bronchoscopes. This technique may however be potentially used more with the exploration of FB in neonatal awake ECMO, which may reduce the risk of ventilator‐induced lung injury [30]. Newer studies on the role of FB in CDH report the successful use of cryoextraction for thrombus removal [31]. Additional preliminary studies in the pediatric population have shown that cryotherapy may also be beneficial in foreign body removal as well as management of pulmonary hemorrhage in ECMO patients [32, 33]. Plastic bronchitis is an overall rare finding, and most common in patients with CHD [34]. In our study, we found a high incidence of bronchial casts in 31.9%. Various treatment options exist with only anecdotal evidence, and include mucolytics, fibrinolytics, corticosteroids, hypertonic saline, and tissue plasminogen activator [35, 36]. Cryotherapy may be beneficial in these cases, as bronchial casts can be difficult to remove, and the risk of significant hemorrhage may be substantial.

Our cohort represents a group of CDH infants with a particularly severe disease burden, reflected by a high rate of FETO and ECMO, type C/D defect, congenital anomalies, and CHD. Despite this, FB in our cohort was safe with a very low complication rate. We did not see any cases of pneumothorax, only one case with FB‐associated bleeding, and no case with postinterventional fever. However, as temperature is regulated in ECMO patients, fever is rarely seen, and therefore, caution in the interpretation of this finding is warranted. Anatomic airway findings were very common in our cohort. In children with bronchopulmonary dysplasia, tracheobronchomalacia has been associated with increased morbidity [37]. It is possible that this finding may be transferred to infants with CDH and variable degrees of chronic lung disease. Our results show a significantly higher mortality in CDH infants with FB. Given our low procedural complication rate, we believe this finding to rather be a reflection of the demonstrated higher disease burden in infants requiring FB. The most common indication for FB in infants with CDH was prolonged and difficult weaning from ECMO and/or ventilation secondary to persistent atelectasis, which may be an additional indicator of CDH disease severity. One problem in the assessment of FB is the lack of a universal definition of parameters reflecting the procedural risks and benefits [15]. Previously mentioned studies have evaluated chest radiographs, different ventilatory and ECMO settings, and paO2 as parameters, with variable results [38]. In our cohort, all of the evaluated parameters failed to show a significant pre‐ and postprocedural difference, although there was a trend towards increased post‐procedural tidal volumes without a significant change in peak inspiratory pressures. Chest radiographs were considered unchanged in most cases. Chest radiographs may not be the modality of choice, as it is a static tool, and the timing of visible potential changes might be variable. Other modalities, that have already been evaluated in the CDH population and that offer continuous monitoring such as lung ultrasound (LUS) and electrical impedance tomography (EIT), may be better alternatives to evaluate the effect of FB [39]. Currently, however, only anecdotal reports on the use of these modalities in the context of FB exist in the literature, and to our knowledge none in the pediatric population [40]. Future prospective studies should evaluate the potential benefit of these modalities in the context of FB in the critically ill neonatal and pediatric population.

The results of our study are limited by its retrospective and single‐center design. In addition, the lack of uniform definitions and provider‐dependent interpretation of indications, findings, and complications of FB limit the comparison of all studies on FB. We hoped to overcome some limitations by providing exact explanations on the individual definitions used in our study, and by reviewing the performed procedures with at least two experienced FB providers.

## 5 Conclusion

In our cohort of critically ill infants with CDH, FB performed by experienced neonatologists proved to be safe with minimal complications and a high diagnostic and potential therapeutic yield. The necessity for FB may be an additional indicator for CDH disease severity. Further research is needed to define uniform assessment metrics of FB and explore continuous monitoring modalities as an alternative to chest X‐rays, such as EIT or LUS in the context of FB in the pediatric population.

## Author Contributions

**Judith Leyens:** conceptualization, investigation, writing – original draft, methodology, validation, visualization, writing – review and editing, project administration, data curation, resources, formal analysis, software, funding acquisition. **Lukas Schroeder:** investigation, writing – review and editing, methodology, validation, conceptualization. **Carmen Salatsch:** conceptualization, investigation, writing – review and editing. **Joachim Schmitt:** conceptualization, investigation, writing – review and editing. **Hemmen Sabir:** conceptualization, investigation, writing – review and editing. **Andreas Mueller:** conceptualization, investigation, methodology,writing – review and editing, resources, supervision. **Florian Kipfmueller:** conceptualization, investigation, writing – review and editing, methodology, project administration, resources, supervision. **Till Dresbach:** conceptualization, investigation, writing – original draft, writing – review and editing, methodology, formal analysis, project administration, data curation, supervision, resources, validation.

## Ethics Statement

This study followed the Declaration of Helsinki guidelines and has been reviewed and approved by the University Hospital of Bonn Institutional Ethics Committee (No. 2024‐435‐BO). Informed consent was waived due to the retrospective study design.

## Guarantor Statement

Judith Leyens takes responsibility for the content of the manuscript, including the data and analysis.

## Conflicts of Interest

The authors declare no conflicts of interest.

## Data Availability

The data that support the findings of this study are available on request from the corresponding author. The data are not publicly available due to privacy or ethical restrictions.

## References

[1] M. R. McGivern , K. E. Best , J. Rankin , et al., “Epidemiology of Congenital Diaphragmatic Hernia in Europe: A Register‐Based Study,” Archives of Disease in Childhood ‐ Fetal and Neonatal Edition 100, no. 2 (2015): F137–F144. 25411443 10.1136/archdischild-2014-306174

[2] N. Patel , P. A. Lally , F. Kipfmueller , et al., “Ventricular Dysfunction Is a Critical Determinant of Mortality in Congenital Diaphragmatic Hernia,” American Journal of Respiratory and Critical Care Medicine 200, no. 12 (2019): 1522–1530. 31409095 10.1164/rccm.201904-0731OC

[3] J. A. Deprest , K. H. Nicolaides , A. Benachi , et al., “Randomized Trial of Fetal Surgery for Severe Left Diaphragmatic Hernia,” New England Journal of Medicine 385, no. 2 (2021): 107–118. 34106556 10.1056/NEJMoa2027030 PMC7613453

[4] A. L. W. Tho , C. P. Rath , J. K. G. Tan , and S. C. Rao , “Prevalence of Symptomatic Tracheal Morbidities After Fetoscopic Endoluminal Tracheal Occlusion: A Systematic Review and Meta‐Analysis,” Archives of Disease in Childhood ‐ Fetal and Neonatal Edition 109, no. 1 (2024): 52–58. 10.1136/archdischild-2023-325525 37419685

[5] P. T. Yu , H. C. Jen , S. Rice‐Townsend , and Y. S. Guner , “The Role of ECMO in the Management of Congenital Diaphragmatic Hernia,” Seminars in Perinatology 44, no. 1 (2020): 151166. 31472951 10.1053/j.semperi.2019.07.005

[6] D. Schramm , N. Freitag , T. Nicolai , et al., “Pediatric Airway Endoscopy: Recommendations of the Society for Pediatric Pneumology,” Respiration 100, no. 11 (2021): 1128–1145. 34098560 10.1159/000517125

[7] A. Young , K. Patel , K. Allen , S. Ghadersohi , M. Rowland , and I. Hazkani , “Flexible and Rigid Bronchoscopy for Critically Ill Children on Extracorporeal Membrane Oxygenation,” The Laryngoscope 134, no. 9 (2024): 4134–4140. 38651446 10.1002/lary.31460

[8] K. Nose , S. Kamata , T. Sawai , et al., “Airway Anomalies in Patients With Congenital Diaphragmatic Hernia,” Journal of Pediatric Surgery 35, no. 11 (2000): 1562–1565. 11083423 10.1053/jpsu.2000.18310

[9] E. Prentice and C. W. Mastropietro , “Flexible Bronchoscopy for Children on Extracorporeal Membrane Oxygenation for Cardiac Failure*,” Pediatric Critical Care Medicine 12, no. 4 (2011): 422–425. 21057355 10.1097/PCC.0b013e3181fe3010

[10] E. A. Rosner , J. L Parker , C. Vandenberg , et al., “Flexible Bronchoscopy in Pediatric Venovenous Extracorporeal Membrane Oxygenation,” Respiratory Care 67, no. 6 (2022): 688–693. 35351825 10.4187/respcare.09243

[11] L. F. Tang and Z. M. Chen , “Fiberoptic Bronchoscopy in Neonatal and Pediatric Intensive Care Units: A 5‐Year Experience,” Medical Principles and Practice 18, no. 4 (2009): 305–309. 19494539 10.1159/000215729

[12] A. Sachdev , N. Gupta , A. Khatri , G. Jha , and G. R. Menon , “Utility and Safety of Flexible Fiberoptic Bronchoscopy in Mechanically Ventilated Children in Pediatric Intensive Care Unit,” Pediatric Pulmonology 57, no. 5 (2022): 1310–1317. 35170875 10.1002/ppul.25863

[13] K. G. Snoek , I. K. M. Reiss , A. Greenough , et al., “Standardized Postnatal Management of Infants With Congenital Diaphragmatic Hernia in Europe: The CDH EURO Consortium Consensus ‐ 2015 Update,” Neonatology 110, no. 1 (2016): 66–74. 27077664 10.1159/000444210

[14] F. Kipfmueller , B. Bo , J. Schmitt , et al., “Percutaneous, Ultrasound‐Guided Single‐ and Multisite Cannulation for Veno‐Venous Extracorporeal Membrane Oxygenation in Neonates,” Pediatric Pulmonology 58, no. 9 (2023): 2574–2582. 37314186 10.1002/ppul.26555

[15] D. Schramm , Y. Yu , A. Wiemers , et al., “Pediatric Flexible and Rigid Bronchoscopy in European Centers—Availability and Current Practice,” Pediatric Pulmonology 52, no. 11 (2017): 1502–1508. 28910517 10.1002/ppul.23823

[16] F. Midulla , J. de Blic , A. Barbato , et al., “Flexible Endoscopy of Paediatric Airways,” European Respiratory Journal 22, no. 4 (2003): 698–708. 14582925 10.1183/09031936.02.00113202

[17] O. Keil and N. Schwerk , “Foreign Body Aspiration in Children – Being Safe and Flexible,” Current Opinion in Anaesthesiology 36, no. 3 (2023): 334–339. 36745076 10.1097/ACO.0000000000001251

[18] D. Kohelet , E. Arbel , and E. S. Shinwell , “Flexible Fiberoptic Bronchoscopy – a Bedside Technique for Neonatologists,” The journal of maternal‐fetal & neonatal medicine: the official journal of the European Association of Perinatal Medicine, the Federation of Asia and Oceania Perinatal Societies, the International Society of Perinatal Obstetricians 24, no. 3 (2011): 531–535. 10.3109/14767058.2010.501123 20617894

[19] K. R. Billings , J. C. Rastatter , K. Lertsburapa , and J. W. Schroeder, Jr. , “An Analysis of Common Indications for Bronchoscopy in Neonates and Findings Over a 10‐Year Period,” JAMA otolaryngology‐‐ head & neck surgery 141, no. 2 (2015): 112–119. 25521999 10.1001/jamaoto.2014.3198

[20] J. De Blic , V. Marchac , and P. Scheinmann , “Complications of Flexible Bronchoscopy in Children: Prospective Study of 1,328 Procedures,” European Respiratory Journal 20, no. 5 (2002): 1271–1276. 12449184 10.1183/09031936.02.02072001

[21] E. M. DeBoer , J. D. Prager , G. S. Kerby , and P. C. Stillwell , “Measuring Pediatric Bronchoscopy Outcomes Using an Electronic Medical Record,” Annals of the American Thoracic Society 13, no. 5 (2016): 678–683. 26816220 10.1513/AnnalsATS.201509-576OC PMC6137899

[22] J. Carlens , J. Fuge , T. Price , et al., “Complications and Risk Factors in Pediatric Bronchoscopy in a Tertiary Pediatric Respiratory Center,” Pediatric Pulmonology 53, no. 5 (2018): 619–627. 29393584 10.1002/ppul.23957

[23] L. Ke , M. Shi , F. Zhang , H. Wu , L. Wu , and L. Tang , “The Clinical Application of Flexible Bronchoscopy in a Neonatal Intensive Care Unit,” Frontiers in Pediatrics 10 (2022): 946579. 36299699 10.3389/fped.2022.946579 PMC9589043

[24] A. Field‐Ridley , “Utility of Flexible Fiberoptic Bronchoscopy for Critically Ill Pediatric Patients: A Systematic Review,” World Journal of Critical Care Medicine 4, no. 1 (2015): 77–88. 25685726 10.5492/wjccm.v4.i1.77 PMC4326767

[25] J. Ibrahim , B. Mahmood , R. DiGeronimo , et al, “Ventilation Strategies During Extracorporeal Membrane Oxygenation for Neonatal Respiratory Failure: Current Approaches Among Level IV Neonatal ICUs,” Critical Care Explorations 4, no. 11 (2022): e0779. 36406885 10.1097/CCE.0000000000000779 PMC9668558

[26] P. P. Kamat , J. Popler , J. Davis , et al., “Use of Flexible Bronchoscopy in Pediatric Patients Receiving Extracorporeal Membrane Oxygenation (ECMO) Support,” Pediatric Pulmonology 46, no. 11 (2011): 1108–1113. 21815274 10.1002/ppul.21480

[27] K. H. Karlson, Jr. , C. B. Pickert , S. M. Schexnayder , and M. J. Heulitt , “Flexible Fiberoptic Bronchoscopy in Children on Extracorporeal Membrane Oxygenation,” Pediatric Pulmonology 16, no. 4 (1993): 215–218. 8265268 10.1002/ppul.1950160402

[28] P. Babhalgaonkar , G. Forster , I. B. Masters , E. Haisz , A. Mattke , and S. Rahiman , “Flexible Fibreoptic Bronchoscopy Is Beneficial in Children on Extracorporeal Membrane Oxygenation Support,” Australian Critical Care 38, no. 1 (2025): 101071, 10.1016/j.aucc.2024.05.008. 38960744

[29] S. R. Hintz , A. M. Sheehan , L. P. Halamek , et al., “Use of Bronchoscopy for Congenital Diaphragmatic Hernia Patients on Extracorporeal Membrane Oxygenation,” Clinical Intensive Care 13, no. 2–3 (2002): 103–108.

[30] J. Costa , D. R. Dirnberger , C. D. Froehlich , C. D. Beaty , M. A. Priest , and M. T. Ogino , “Awake Neonatal Extracorporeal Membrane Oxygenation,” ASAIO Journal 66, no. 5 (2020): e70. 31335364 10.1097/MAT.0000000000001029

[31] J. J. Brewington , D. T. Benscoter , C. A. Torres‐Silva , et al., “Flexible Bronchoscopic Thrombus Cryoextraction in a Neonate on Extracorporeal Membrane Oxygenation,” American Journal of Respiratory and Critical Care Medicine 203, no. 5 (2021): 633–635. 33095995 10.1164/rccm.202007-2817LE PMC7924573

[32] A. Green , N. Puri , M. Kouch , et al., “Cryotherapy: A Safe Approach to Pulmonary Hemorrhage During VV‐ECMO,” Journal of Investigative Medicine High Impact Case Reports 10 (2022): 23247096221074590. 35152803 10.1177/23247096221074590 PMC8848090

[33] D. Schramm , N. Freitag , K. Kötz , et al., “Cryotherapy in the Paediatric Airway: Indications, Success and Safety,” Respirology 27, no. 11 (2022): 966–974. 36054726 10.1111/resp.14353

[34] T. Jasinovic , F. K. Kozak , J. P. Moxham , et al., “Casting a Look at Pediatric Plastic Bronchitis,” International Journal of Pediatric Otorhinolaryngology 79, no. 10 (2015): 1658–1661. 26250441 10.1016/j.ijporl.2015.07.011

[35] S. S. Manna , “Treatment of Plastic Bronchitis in Acute Chest Syndrome of Sickle Cell Disease With Intratracheal rhDNAse,” Archives of Disease in Childhood 88, no. 7 (2003): 626–627. 12818912 10.1136/adc.88.7.626 PMC1763153

[36] T. B. Do , J. M. Chu , F. Berdjis , and N. G. Anas , “Fontan Patient With Plastic Bronchitis Treated Successfully Using Aerosolized Tissue Plasminogen Activator: A Case Report and Review of the Literature,” Pediatric Cardiology 30, no. 3 (2009): 352–355. 19005718 10.1007/s00246-008-9312-2

[37] E. B. Hysinger , N. L. Friedman , M. A. Padula , et al., “Tracheobronchomalacia Is Associated With Increased Morbidity in Bronchopulmonary Dysplasia,” Annals of the American Thoracic Society 14, no. 9 (2017): 1428–1435. 28622012 10.1513/AnnalsATS.201702-178OC PMC5711403

[38] C. Yan , Y. Hu , G. Qiu , X. Gong , and D. Elda , “The Clinical Safety and Efficacy of Flexible Bronchoscopy in a Neonatal Intensive Care Unit,” Experimental and Therapeutic Medicine 20, no. 5 (2020): 1. 32973944 10.3892/etm.2020.9223 PMC7507083

[39] L. Schroeder , F. Kipfmueller , B. Hentze , et al., “Evaluation of Regional Ventilation Distributions in Newborns With Congenital Diaphragmatic Hernia,” American Journal of Respiratory and Critical Care Medicine 209, no. 5 (2024): 601–606. 38047881 10.1164/rccm.202305-0797LE

[40] A. Vianello , F. Lionello , and G. Guarnieri , “Electrical Impedance Tomography Used During Bronchoscopy in a Patient With Aspiration Pneumonia,” Archivos de bronconeumología 59, no. 5 (2023): 332. 36803940 10.1016/j.arbres.2023.02.002

